# SSRP1 affects the growth and apoptosis of gastric cancer cells through AKT pathway

**DOI:** 10.5937/jomb0-33374

**Published:** 2022-02-02

**Authors:** Guohua Jin, Ruihong Zhao, Jianguang Zhang, Tingting Cao, Tongyu Tang

**Affiliations:** 1 First Hospital of Jilin University, Department of Gastroenterology, Changchun, Jilin, China

**Keywords:** SSRP1, gastric cancer, proliferation, apoptosis, SSRP1, rak želuca, proliferacija, apoptoza

## Abstract

**Background:**

We aimed to determine the SSRP1's potential influence on the apoptosis and proliferation of gastric cancer (GC) cells and its regulatory mechanism.

**Methods:**

SSRP1 expression in GC cells and tissues was detected via quantitative reverse transcription-polymerase chain reaction (qRT-PCR). The interrelation between clinicopathological characteristics of GC patients and SSRP1 expression was analysed via x2 test, and the correlation between SSRP1 expression and overall survival rate was analysed using Kaplan-Meier survival analysis. After the knockdown of SSRP1 in AGS cells, the SSRP1 expression, colony formation ability, cell viability, cell cycle changes, apoptosis rate, and migration and invasion ability were detected through qRT-PCR, colony formation assay, CCK8 assay, flow cytometry and transwell test, respectively. Finally, the effects of down-regulation of SSRP1 on the expressions of phosphorylated-protein kinase B (p-AKT), B-cell lymphoma-2 (Bcl-2) and Bcl-2 associated X protein (Bax) were explored using Western blotting.

**Results:**

SSRP1 displayed a high expression in GC cells and tissues. SSRP1 expression was closely interrelated to the TNM stage, lymph node metastasis and tumour size. The survival rate of patients was markedly shorter in the high expression group than in the lower expression group. After the knockdown of SSRP1 in cells, the viability and colony formation ability of AGS cells were inhibited. In addition, the cell ratio in the G1 phase was increased, while that in the S phase declined, and the cell invasion and migration were obviously weakened. It was found from Western blotting that the knockdown of SSRP1 could evidently suppress the protein levels of Bcl-2 and p-AKT but promote the protein expression of Bax, indicating that silencing SSRP1 can inhibit the proliferative capacity and increase the number of GC cells through inactivating the AKT signalling pathway.

**Conclusions:**

SSRP1 rose up in GC tissues and cells. Reduction of SSRP1 can inhibit the proliferative capacity and increase the number of GC cells through inactivating the AKT signalling pathway.

## Abstract (sr)

**Uvod:**

Naš cilj je bio da otkrijemo potencijalni uticaj SSRP1 na apoptozu i proliferaciju ćelija raka želuca (GC) i njegov regulatorni mehanizam.

**Metode:**

Ekspresija SSRP1 u GC ćelijama i tkivima je detektovana kvantitativnom lančanom reakcijom polimeraze reverzne transkripcije (qRT-PCR). Odnos između kliničkopatoloških karakteristika pacijenata sa GC i ekspresije SSRP1 je analiziran pomoću Hi-kvadratnog testa (x2), a korelacija između ekspresije SSRP1 i ukupne stope preživljavanja pomoću Kaplan-Meier analize preživljavanja. Nakon obaranja SSRP1 u ćelijama AGS, ekspresija SSRP1, sposobnost formiranja kolonije, vitalnost ćelija, promene ćelijskog ciklusa, stopa apoptoze i sposobnost migracije i invazije su otkriveni pomoću qRT-PCR, testa formiranja kolonije, CCK8 testa, protočne citometrije i transvel testa, redom. Konačno, efekti smanjenja SSRP1 na ekspresije fosforilisane protein kinaze B (p-AKT), B-ćelijskog limfoma2 (Bcl-2) i Bcl-2 pridruženog Ks proteina (Bax) su ispitani pomoću Vestern blota.

**Rezultati:**

SSRP1 je pokazao visoku ekspresiju u GC ćelijama i tkivima. Ekspresija SSRP1 je bila usko povezana sa stadijumom TNM, metastazama u limfnim čvorovima i veličinom tumora. Stopa preživljavanja pacijenata je bila znatno kraća u grupi sa visokom ekspresijom nego u grupi sa nižom ekspresijom. Nakon obaranja vrednosti SSRP1 u ćelijama, došlo je do inhibicije vitalnosti i sposobnosti formi ranja kolonija AGS ćelija. Pored toga, povećana je elijska stopa u G1 fazi, dok je u S fazi opala, dok su invazija i migracija ćelija očigledno oslabljene. Vestern blot metodom je utvđeno da bi obarenje SSRP1 moglo očigledno potisnuti nivo proteina Bcl-2 i p-AKT, ali bi promovisalo ekspresiju proteina Bak, ukazujući da smanjenje SSRP1 može inhibirati proliferativni kapacitet i povećati broj GC ćelija kroz inaktivaciju AKT signalnog puta.

**Zaključak:**

SSRP1 je porastao u tkivima i ćelijama GC. Smanjenje SSRP1 može inhibirati proliferativni kapacitet i povećati broj GC ćelija kroz inaktivaciju AKT signalnog puta.

## Introduction

Gastric cancer (GC) is a frequently seen malignancy seriously threatening human health. According to the latest statistics, the morbidity rate of GC ranks 4th among all malignant tumours in the world, and its mortality rate has jumped to 3rd place [1] [2]. The 5-year survival rate of GC patients in China has dramatically risen with the early gastroscopy and prompt surgical intervention in recent years [3]. However, the 5-year survival rate of advanced GC remains low [4]. Therefore, further uncovering the pathogenesis of GC for preventing the occurrence of GC and improving the prognosis is of great significance.

Structure-specific recognition protein 1 (SSRP1) was originally identified as a high-mobility group 1 (HMG1)-related DNA-binding protein in 1991, and its biological functions can be attributed to its HMG domain [5]. SSRP1, a subgroup of histone chaperones, boosts chromatin transcription (FACT) complex and regulates most processes associated with chromatins, such as replication, DNA repair, and transcription [6] [7] [8] [9]. It is believed that SSRP1 dramatically influences the development and occurrence of tumours, and its expression is upregulated in such tumours as hepatocellular cancer [10], colorectal cancer [11], nasopharyngeal cancer [12] and glioma [13]. Knockdown of SSRP1 *in vitro* can suppress the proliferative capacity of glioma U251 and U87 cells by inactivating the MAPK pathway [13] and reducing the growth of NSCLC cells [14]. Nevertheless, the biological role and mechanism of SSRP1 in gastric cancers remain unclear.

To clarify SSRP1's function in GC and its mechanism of action, therefore, SSRP1 expression in GC cells and tissues was explored through a series of functional assays, and its effects on the proliferative capacity, apoptosis, invasion and migration of GC cells via the protein kinase B (AKT) pathway were determined, thereby providing new ideas for the targeted clinical treatment of GC.

## Materials and Methods

### Patients and tissue specimens

A total of 40 paired GC specimens were surgically resected in our hospital. The patients had complete clinicopathological data and follow-up data. Specimens receiving 5-min fast freezing in liquid nitrogen were reserved at -80°C. The present research was reviewed and obtained approval of the Hospital Medical Ethics Committee, and the written informed consent was obtained from all participants.

### Cell culture and transfection

AGS, MGC-803, FU97, BGC-823, GES-1 and SGC-7901 cells were cultured in 1640 medium from Invitrogen (Carlsbad, CA, USA) containing 10% fetal bovine serum (FBS) provided by GIBCO/BRL (Rockville, MD, USA), 100 U/mL penicillin and 100 mg/mL streptomycin from Beyotime (Shanghai, China) under 5% CO_2_ at 37°C. AGS cells undergone siRNA (GenePharma, Suzhou, China) or NC transfection using Lipofectamine 2000 transfection reagent (Invitrogen, Carlsbad, CA, USA) as per the guidance of the manufacturer si-SSRP1 sense: 5'-GCCAUGU-CUACAAGUAUGATT-3', and antisense: 5'-UCAUA-CUUGUAGACAUGGCTT-3'.

### Quantitative reverse transcription-polymerase chain reaction (qRT-PCR)

RNAs underwent RT into cDNAs on GoScript RT System (Promega, Madison, WI, USA) by an All-in-One miRNA RT kit (GeneCopoeia, Rockville, MD, USA). Then SYBR Green human miRNA kit (GeneCopoeia, Rockville, MD, USA) and GoTaq qPCR Master Mix (Promega, Madison, WI, USA) were utilised for qRT-PCR. The procedures of thermal cycler were set below: 95°C for 10 min, (95°C for 30 s) × 40 cycles, primer annealing at 55°C for 30 s and 72°C for 30 s. Each assay was repeated for 3 times.

### Western blotting

The cells were harvested and lysed with 1× cell lysate from Cell Signaling Technology (Danvers, MA, USA) with phenylmethylsulfonyl fluoride (PMSF) (1 mmol/L) at 4°C for 30-60 min. Subsequent to 10-min centrifugation at 12,000 g, the cytoplasmic protein samples were obtained, and bicinchoninic acid (BCA) protein test kit from Thermo Fisher Scientific (Waltham, MA, USA) was utilised for measurement of protein concentration. Then, total protein samples (20-50 μg) were isolated via 10-12% sodium dodecyl sulphate-polyacrylamide gel electrophoresis (SDS-PAGE) and transferred onto a polyvinylidene fluoride (PVDF) membrane from Millipore (Billerica, MA, USA). The membrane was blocked by 5% skim milk at room temperature for 2 h, and undergone primary antibody (1:1,000) incubation at 4°C nightlong. On the following day, TBS with 0.05% (v/v) Tween-20 (TBST) was utilised for rinsing the membrane, and it received 2-h incubation with secondary antibodies jugated by horseradish peroxidase (1:5,000, Lianke Biotech Co., Ltd., Hangzhou, China) at indoor temperature. Following rinsing, the membrane was observed using an electrochemiluminescence (ECL) assay kit (PerkinElmer, Inc., Rockford, IL, USA) and Tanon 5500 gel imaging system from Tanon Science & Technology (Shanghai, China).

### Detection of proliferative activity via cell counting kit-8 (CCK8) test

Cells undergoing transfection were paved onto a ninety-six-well plate (1000 cells/well), and cell proliferation was measured once a day as per the manufacturer's instructions. In a word, each well was added with 10 µL of CCK8 solution (KeyGen BioTECH, Nanjing, China) for 2-h cell incubation at 37°C. Finally, the solution was assessed at 450 nm via spectrophotometry. The assay was repeated for 3 times in each group.

### Colony formation experiment

Cells receiving transfection were paved onto a 6-well plate (200 cells/well), cultivated for 2 weeks, fixed with paraformaldehyde and dyed by Giemsa dye. Then formed colonies were counted, and the colony formation rate in each plate was calculated. The assay was repeated for 3 times in each group.

### Examination of cell cycle and apoptosis rate via flow cytometry

Following at least two hours of immobilisation in cold methanol, cells received RNase A/propidium iodide (PI) solution incubation. After that, the FACSCalibur system from Becton-Dickinson (Franklin Lakes, NJ, USA) was utilised for distribution assessment of cell cycle, and the proportion of cells in varying phases was detected via Modfit 2.0 software. Flow cytometry and Annexin V/PI dying (Life Technologies, CA, USA) were implemented for apoptotic cell determination. This test was implemented thrice, and findings were presented as mean ± SD. The proportion of apoptotic cells was analysed using FlowJo7.6.1 software.

### Transwell assay

The motility of tumour cells in vitro was assessed using chambers for Transwell migration from Corning (Corning, NY, USA) and those for invasion from BD Biosciences (Franklin Lakes, NJ, USA). Briefly, 200,000 cells containing siRNA or not were added into medium supplemented with 2% FBS, and the lower chamber was added with the medium with 10% FBS for 48 h. Then cells migrating/invading the base of the upper membrane were dyed with 0.1% crystal violet dye. Next, the cells were counted in five fields stochastically chosen using a microscope.

### Statistical analysis

Data were processed via Statistical Product and Service Solutions (SPSS) 20.0 software (IBM, Armonk, NY, USA). Metrical data were displayed by mean ± SD (x̄ ± SD). Intergroup differences were processed through t-test and one-way analysis of variance (ANOVA). Comparison of enumeration data was implemented using χ^2^ test. Survival curves were plotted using the Log-rank test (Kaplan-Meier method). P<0.05 reflected statistically significant differences.

## Results

### SSRP1 displayed a high expression in GC tissues and cells

**Figure 1:**
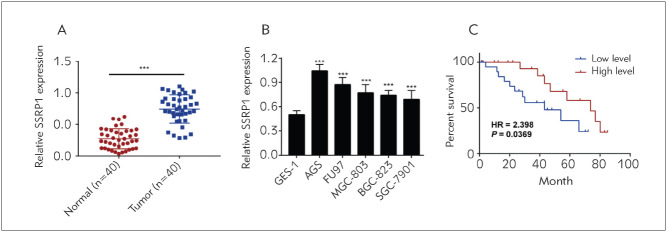
SSRP1 displays high expressions in GC cells and tissues (A) It is found from qRT-PCR that SSRP1 expression obviously rose in 40 cases of GC tissues relative to that in para-carcinoma normal tissues<br>(B) The expression of SSRP1 is detected in GES-1 as well as AGS, FU97, MGC-803, BGC-823 and SGC-7901<br>(C) Kaplan-Meier survival analysis. Patients had a lower survival rate in the high-expression group relative to that in the low-expression group

It was found from qRT-PCR that SSRP1 expression rose up in 40 GC tissues compared with that in para-carcinoma normal ones (Figure 1A). SSRP1 expression was further detected in GES-1 as well as AGS, FU97, MGC-803, BGC-823 and SGC-7901. It was discovered that SSRP1 expression rose in GC cell lines, and it showed the highest value in AGS cell lines, so AGS cell lines were selected for subsequent knockdown assay (Figure 1B). According to the median expression of SSRP1, the patients were allocated into high-and low-expression groups, and the interrelations of SSRP1 expression with clinical and pathological features of patients was further analysed. The results revealed that the patients in the high-expression group displayed a larger tumour size and advanced TNM stage, and are often accompanied by lymph node metastasis, showing statistically significant differences (P<0.05) (Table 1). Besides, the Kaplan-Meier survival curves were analysed. It was confirmed that patients in the high-expression group had a lower survival rate relative to that in the lowexpression group (HR=2.3980, P=0.0369), indicating that SSRP1 predicts the unfavourable prognosis of GC patients. The above findings demonstrate that SSRP1 is highly expressed in GC patients, and it can affect the TNM stage, lymph node metastasis and tumour size of GC patients, indicating a poor prognosis.

**Table 1:** Relations of SSRP1 expression with clinical and pathological features in GC patients (n=40)

Clinicopathologic<br>features	Number<br>of cases	SSRP1 expression		P-value
		Low<br>(n=20)	High<br>(n=20)	
Age (years)				0.752
≥60	19	10	9	
>60	21	10	11	
Gender				0.749
Male	23	11	12	
Female	17	9	8	
Tumour size				0.022*
≤5CM	15	11	4	
>5CM	25	9	16	
TNM stage				0.027*
I+II	19	13	6	
III+IV	21	7	14	
Tumour<br>differentiation				0.113
Well/Moderate	21	13	8	
Poor	29	17	12	
Lymph node<br>metastasis				0.004*
Absent	19	14	5	
Present	21	6	15	
Distant metastasis				
Positive		11	8	0.342
Negative			9	12

### Knockdown of SSRP1 inhibited GC cell proliferation

**Figure 2:**
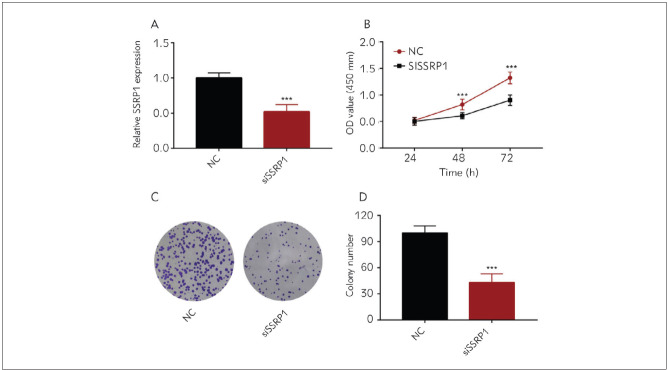
Decreasing SSRP1 impedes GC cells to proliferate (A) QRT-PCR findings reveal that the mRNA expression of SSRP1 is obviously suppressed by knockdown of SSRP1 at 48 h after AGS cells are transfected with siRNA<br>(B) The results of the CCK8 assay show that the AGS cell viability declines after the knockdown of SSRP1<br>(C) Colony formation assay findings show that the colony formation ability of AGS cells becomes weaker after the knockdown of SSRP1

To explore SSRP1's role in GC development, AGS cell lines were further selected for knockdown assay and whether changes in SSRP1 affect GC cells was explored. QRT-PCR findings ascertained that SSRP1 mRNA expression dropped obviously after AGS cells were transfected with si-SSRP1 (Figure 2A). Then SSRP1's impact on AGS cell activity was assessed. It was unveiled from the CCK8 assay showed that cell activity was obviously weakened after knockdown of SSRP1 in contrast to that in the control group (Figure 2B). According to the colony formation assay, the capacity of AGS cells treated with siRNA to form colonies also became weaker (Figure 2C), implying that knocking down SSRP1 impedes GC cells to proliferate.

### SSRP1 influenced GC cells to proliferate and their apoptosis by impeding the AKT signaling pathway

**Figure 3:**
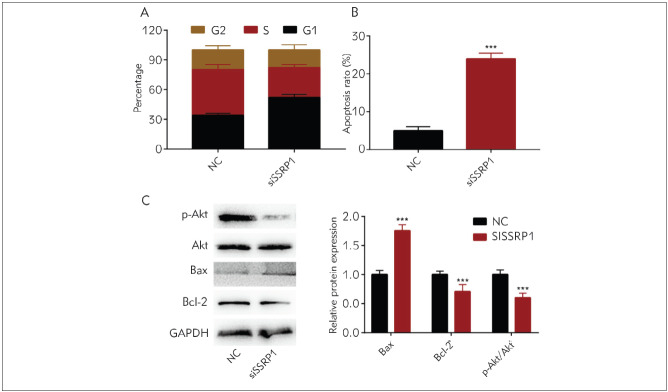
SSRP1 affects GC cells to proliferate and their apoptosis via impeding the AKT signalling pathway (A) Flow cytometry findings manifest that cell proportion rises in the G1 phase and declines in the S phase, and no significant changes are found in cell proportion in the G2 phase<br>(B) Flow cytometry findings manifest that the apoptosis rate rises markedly after the knockdown of SSRP1<br>(C & D) Western blotting results show that knockdown of SSRP1 can reduce the expressions of p-AKT and Bcl-2 proteins but increase the protein expression of Bax

SSRP1's impacts on the cycle and apoptosis of AGS cells were explored using flow cytometry. As shown in Figure 3A, at 48 h after treatment of AGS cells with siRNA, cell proportion became larger in the G1 phase but smaller in the S phase, confirming that knockdown of SSRP1 can inhibit the transition of the G1/S phase. In addition, it was found from flow cytometry that the apoptosis rate rose markedly after the knockdown of SSRP1 (Figure 3B). As a classical signal transduction pathway, the AKT pathway is implicated in apoptosis and proliferation. Furthermore, the results of Western blotting manifested that knockdown of SSRP1 could suppress the protein expressions of phosphorylated AKT (p-AKT) and B-cell lymphoma-2 (Bcl-2), but evidently promote Bax protein expression (Figure 3C & Figure 3D), indicating that silencing SSRP1 may inhibit GC cells from proliferating and promoting their apoptosis through the AKT signalling pathway.

### Down-regulation of SSRP1 suppressed GC cells to migrate and invade

**Figure 4:**
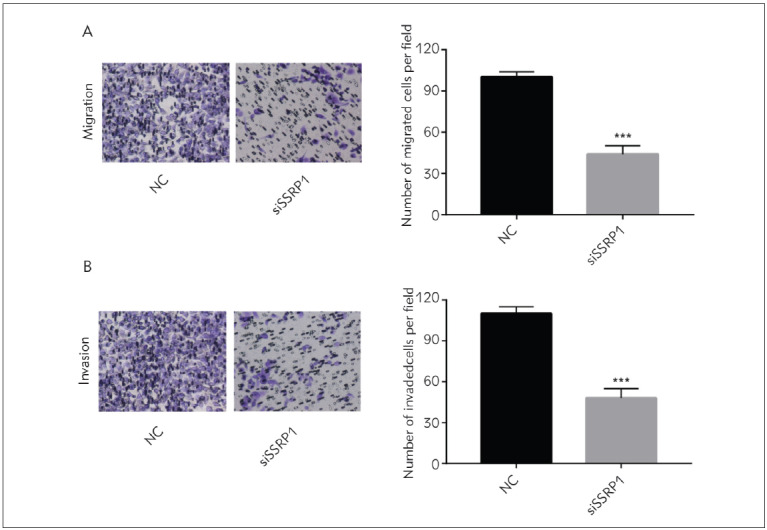
Down-regulation of SSRP1 suppresses GC cells to migrate and invade (A & B) Transwell assay findings show that after reducing SSRP1, the AGS cell migration and invasion capacities remarkably decline

As confirmed above, down-regulation of SSRP1 can impede GC cells to proliferate and promote their apoptosis. Then, SSRP1's influences on the GC cells' abilities to migrate and invade were further explored. Transwell assay findings showed that after SSRP1 knockdown, the cell migration and invasion ability remarkably declined (Figure 4A & Figure 4B). Thus, it can be inferred that decreasing SSRP1 can impair the migration and invasion capacities of GC cells.

## Discussion

It is reported that SSRP1, a subunit of histone chaperone protein FACT, can destroy the nucleosomes and histone substitutes [15] [16]. Moreover, it is implicated in DNA damage repair and replication as well as cell proliferation and apoptosis [14] [17]. In the current research, it was disclosed that SSRP1 rose in GC tissues and cells, consistent with previous studies on cancers, including NSCLC [14], breast cancer [18] [19] and liver cancer [10]. The survival rate of patients was lower in the high-expression group than in the low-expression group, indicating that highly expressed SSRP1 predicts the unfavourable prognosis of GC patients. Additionally, the results of a series of *in vitro* experiments confirmed that knockdown of SSRP1 could inhibit the vitality, colony formation ability and cell cycle transition of GC cells and induce apoptosis, and may also suppress cell migration and invasion.

SSRP1 is a key regulatory factor for keeping normal DNA replication because it is related to MCM helicase and can promote the unwinding activity of MCM helicase DNA on the nucleosome template [20] [21]. Breaking the FACT-MCM complex can hinder the beginning of DNA replication. Inhibiting SSRP1 can suppress cells to grow and hamper the S-phase cell cycle progression owing to suppression on the replication fork advancement [20]. Proliferation signals accompanied by enhanced DNA replication are the markers for cancer cells [22]. In this study, it was found that decreasing SSRP1 repressed the viability and colony formation ability of GC cells, which is a characteristic feature of GC cells.

In this study, it was confirmed that SSRP1 played a role in regulating GC cells to proliferate and regulated their cycle, apoptosis, invasion, and migration capacities. It was unveiled that knockdown of SSRP1 could lead to cell cycle arrest and induce apoptosis. Moreover, after the knockdown of SSRP1, the protein expression of Bcl-2 could be inhibited, whereas that of Bax could be promoted. During the progression of GC, metastasis is the fatal event, so early diagnosis of highly metastatic primary tumours is of important significance for the treatment and prognosis of patients. In this study, the findings revealed that SSRP1 knockdown impeded GC cells to migrate and invade. In addition, it is reported that SSRP1 is implicated in the classical PI3K/AKT signalling pathway and affects the apoptosis and proliferation of colon cancer cells via the AKT pathway [23]. As the main signalling pathway in the downstream of many growth factor receptors, a classical signalling pathway related to cell apoptosis and survival, the PI3K/AKT pathway, is the most active signalling pathway in human tumours. Moreover, the PI3K/AKT pathway facilitates the proliferation and malignant transformation and impedes tumour cell apoptosis by phosphorylating PI3K and AKT proteins [24] [25]. Serine/threonine kinase AKT, classified into AKT1-3, is a key PI3K signal transduction factor in mammals. Numerous studies have unravelled that the PI3K/AKT pathway can promote GC cells to proliferate and invade [26] [27]. In this study, therefore, the involvement of the AKT pathway in alterations of GC cell proliferation and apoptosis following interference with SSRP1 was explored. The findings manifested that knockdown of SSRP1 reduced the expression of p-AKT, indicating that SSRP1 regulates GC cell proliferation, cycle, apoptosis, invasion and migration via activating the AKT pathway.

## Conclusion

Knockdown of SSRP1 affects GC cells to proliferate and their apoptosis through the AKT pathway, which provides a new possible therapeutic strategy and diagnostic target for GC.

## Dodatak

### Conflict of interest statement

The authors reported no conflict of interest regarding the publication of this article.

## References

[1] Liang D, Liang S, Jin J, Li D J, Shi J, He Y (2017). Gastric cancer burden of last 40 years in North China (Hebei Province): A population-based study. Medicine (Baltimore).

[2] Siegel R L, Miller K D, Jemal A (2017). Cancer statistics, 2017. CA Cancer J Clin.

[3] Hamashima C, Shabana M, Okada K, Okamoto M, Osaki Y (2015). Mortality reduction from gastric cancer by endoscopic and radiographic screening. Cancer Sci.

[4] Wu H, Zhu R, Li Y, Wu X, Xie R, Li H, Wang H, Zhang H, Xiao H, Chen H, Zhen H, Zhao K, Yang X, Xie M, Tuo B, 4. di Lianjun, (2017). Multi-disciplinary team for early gastric cancer diagnosis improves the detection rate of early gastric cancer. BMC Gastroenterol.

[5] Röttgers K, Krohn N M, Lichota J, Stemmer C, Merkle T, Grasser K D (2000). DNA-interactions and nuclear localisation of the chromosomal HMG domain protein SSRP1 from maize. Plant J.

[6] Kumari A, Mazina O M, Shinde U, Mazin A V, Lu H (2009). A role for SSRP1 in recombination-mediated DNA damage response. J Cell Biochem.

[7] Birch J L, Tan N, Panov K I, Panova T B, Andersen J S, Owen-Hughes T A, Russell J, Lee S, Zomerdijk J C B M (2009). FACT facilitates chromatin transcription by RNA polymerases I and III. EMBO J.

[8] Orphanides G, Wu W H, Lane W S, Hampsey M, Reinberg D (1999). The chromatin-specific transcription elongation factor FACT comprises human SPT16 and SSRP1 proteins. Nature.

[9] Zhang W, Zeng F, Liu Y, Shao C, Li S, Lv H, Shi Y, Niu L, Teng M, Li X (2016). Crystal Structure of Human SSRP1 Middle Domain Reveals a Role in DNA Binding. Sci Rep.

[10] Ding Q, He K, Luo T, Deng Y, Wang H, Liu H, Zhang J, Chen K, Xiao J, Duan X, Huang R, Xia Z, Zhou W, He J, Yu H, Jiao X, Xiang G (2016). SSRP1 Contributes to the Malignancy of Hepatocellular Carcinoma and Is Negatively Regulated by miR-497. Mol Ther.

[11] Wu W, He K, Guo Q, Chen J, Zhang M, Huang K, Yang D, Wu L, Deng Y, Luo X, Yu H, Ding Q, Xiang G (2019). SSRP1 promotes colorectal cancer progression and is negatively regulated by miR-28-5p. J Cell Mol Med.

[12] Gao L, Xiong X (2018). MiR-223 inhibits the proliferation, invasion and EMT of nasopharyngeal carcinoma cells by targeting SSRP1. Int J Clin Exp Pathol.

[13] Liao J, Tao X, Ding Q, Liu J, Yang X, Yuan F E, Yang J, Liu B, Xiang G, Chen Q (2017). SSRP1 silencing inhibits the proliferation and malignancy of human glioma cells via the MAPK signaling pathway. Oncol Rep.

[14] Dermawan J K T, Gurova K, Pink J, Dowlati A, Narla G, Sharma N, Stark G R, de Sarmishtha, (2014). Quinacrine Overcomes Resistance to Erlotinib by Inhibiting FACT, NF-cB, and Cell-Cycle Progression in Non-Small Cell Lung Cancer. Mol Cancer Ther.

[15] Takahata S, Yu Y, Stillman D J (2009). FACT and Asf1 Regulate Nucleosome Dynamics and Coactivator Binding at the HO Promoter. Mol Cell.

[16] Tsunaka Y, Fujiwara Y, Oyama T, Hirose S, Morikawa K (2016). Integrated molecular mechanism directing nucleosome reorganization by human FACT. Genes Dev.

[17] Gao X J, Feng J X, Zhu S, Liu X H, Tardieux I, Liu L X (2014). Protein Phosphatase 2C of Toxoplasma Gondii Interacts with Human SSRP1 and Negatively Regulates Cell Apoptosis. Biomed Environ Sci.

[18] Koman I E, Commane M, Paszkiewicz G, Hoonjan B, Pal S, Safina A, Toshkov I, Purmal A A, Wang D, Liu S, Morrison C, Gudkov A V, Gurova K V (2012). Targeting FACT Complex Suppresses Mammary Tumorigenesis in Her2/neu Transgenic Mice. Cancer Prev Res (Phila).

[19] Fleyshman D, Prendergast L, Safina A, Paszkiewicz G, Commane M, Morgan K, Attwood K, Gurova K (2017). Level of FACT defines the transcriptional landscape and aggressive phenotype of breast cancer cells. Oncotarget.

[20] Abe T, Sugimura K, Hosono Y, Takami Y, Akita M, Yoshimura A, Tada S, Nakayama T, Murofushi H, Okumura K, Takeda S, Horikoshi M, Seki M, Enomoto T (2011). The Histone Chaperone Facilitates Chromatin Transcription (FACT) Protein Maintains Normal Replication Fork Rates. J Biol Chem.

[21] Zeng S X, Li Y, Jin Y, Zhang Q, Keller D M, Mcquaw C M, Barklis E, Stone S, Hoatlin M, Zhao Y, Lu H (2010). Structure-Specific Recognition Protein 1 Facilitates Microtubule Growth and Bundling Required for Mitosis. Mol Cell Biol.

[22] Hanahan D, Weinberg R A (2011). Hallmarks of Cancer: The Next Generation. Cell.

[23] Wang Q, Jia S, Jiao Y, Xu L, Wang D, Chen X, Hu X, Liang H, Wen N, Zhang S, Guo B, Zhang L (2019). SSRP1 influences colorectal cancer cell growth and apoptosis via the AKT pathway. Int J Med Sci.

[24] Laplante M, Sabatini D M (2012). mTOR Signaling in Growth Control and Disease. Cell.

[25] Mabuchi S, Kuroda H, Takahashi R, Sasano T (2015). The PI3K/AKT/mTOR pathway as a therapeutic target in ovarian cancer. Gynecol Oncol.

[26] Lu Y, Li L, Wu G, Zhuo H, Liu G, Cai J (2019). Effect of PI3K/Akt Signaling Pathway on PRAS40Thr246 Phosphorylation in Gastric Cancer Cells. Iran J Public Health.

[27] Hao N B, Tang B, Wang G Z, Xie R, Hu C J, Wang S Z, Wu Y, Liu E, Xie X, Yang S (2015). Hepatocyte growth factor (HGF) upregulates heparanase expression via the PI3K/Akt/NF-cB signaling pathway for gastric cancer metastasis. Cancer Lett.

